# Host Factors Potentially Contributing to Increased Susceptibility in Certain Layer Chicken Lines

**DOI:** 10.3390/cimb48040359

**Published:** 2026-03-29

**Authors:** Yiqun Chen, Junlong Xiong, Yicheng Wang, Siyue Huang, Mingyu Fan, Heng Yang, Zhiqiang Hu, Jingang Zhao, Chaoyun Yang, Jun Li, Jing Wang, Zengwen Huang

**Affiliations:** 1 College of Animal Science, Xichang University, Xichang 615000, China; xcc20250039@xcc.edu.cn (Y.C.); 15984982742@163.com (Y.W.); 17381428008@163.com (S.H.); zhiqianghu0624@xcc.edu.cn (Z.H.); zjingang2022@163.com (J.Z.); chaoyuny@yeah.net (C.Y.); lijun@xcc.edu.cn (J.L.); slnee.5703@163.com (J.W.); 2 College of Veterinary Medicine, South China Agricultural University, Guangzhou 510642, China; 15528133512@163.com; 3 College of Veterinary Medicine, Northwest A&F University, Yangling 712100, China; keepitup9968@126.com; 4 College of Veterinary Medicine, Southwest University, No. 2, Tiansheng Street, Chongqing 400715, China; yhuv2013@sina.com

**Keywords:** avian influenza virus, layer, broiler, susceptibility, hormone

## Abstract

Avian influenza (AI) continues to threaten global poultry production, with accumulating evidence suggesting that certain commercial layer lines may exhibit increased susceptibility under specific experimental conditions compared with broiler chickens. This narrative review synthesizes published experimental infection studies identified through a comprehensive PubMed search, focusing on low pathogenic H9N2 and highly pathogenic H5N1, H5N2, H7N7, and H7N9 viruses. Although bird age and production stage varied across studies, consistent disparities in immune regulation and viral replication dynamics have been reported. We critically evaluate host determinants underlying these differences—including microRNAs, major histocompatibility complex polymorphisms, sialic acid receptor distribution, gut microbiota, and hormonal influences—and integrate findings across viral subtypes and pathogenicity classes to inform breed-tailored vaccination, nutritional, and therapeutic strategies.

## 1. Introduction

Influenza A virus is a multi-host pathogen that significantly impacts the health of wildlife, domestic animals, and humans [1]. Wild birds, particularly those in the orders Anseriformes and Charadriiformes, serve as the primary reservoirs for avian influenza virus (AIV), which is classified into highly pathogenic (HPAIV) and low pathogenic (LPAIV) strains [2]. AIV transmission from these reservoirs to domestic poultry leads to severe socioeconomic consequences and poses zoonotic risks [3], with subtypes such as H5N1, H7N9, and H9N2 confirmed to infect humans [4]. Among these, the H5 and H7 subtypes are particularly devastating [5]. Notably, the H7 viruses infect a wide range of species [6]. The H7N9 virus, for instance, emerged as a low pathogenic strain in 2013 [7] but evolved into high pathogenicity by 2017 [8], facilitating reassortment and the generation of novel lethal viruses [9]. Cross-species transmission plays a crucial role in the ecology of these viruses [10], which have caused massive poultry losses alongside H5 subtypes.

Host responses to infectious diseases are generally characterized by significant heterogeneity [11]. While comparative studies in mammals have well-documented sex-based differences in susceptibility—such as the variance in morbidity between males and females infected with influenza or other viruses [12,13,14,15]—there is a notable lack of comprehensive literature addressing breed-based differences in chickens. This gap exists despite the fact that modern poultry farming relies on two highly specialized breeds evolved through decades of selective breeding: layer-type chickens and broiler-type chickens. These distinct genetic backgrounds significantly influence their physiological performance and immunological responses [16,17,18]. Research indicates that selection for rapid growth in broilers may trade off with immune responses, whereas layers prioritize sustained immune function [18,19,20]. Consequently, studies using RNA sequencing have identified critical genes and pathways (e.g., NF-κB and JAK/STAT) associated with disease resistance [21].

Emerging epidemiological and experimental evidence suggests a marked disparity in AIV susceptibility profiles among different poultry phenotypes. In the context of this review, “susceptibility” is broadly defined as a multifaceted outcome encompassing higher mortality rates, increased viral replication and shedding, broader tissue tropism, and severe production losses (such as precipitous drops in egg laying). It is important to note that while direct, head-to-head experimental comparisons between commercial layers and broilers remain limited, a wealth of knowledge can be extrapolated from independent studies and diverse comparative models (e.g., commercial layers versus waterfowl, or highly susceptible lines versus indigenous resistant breeds). By synthesizing these disparate datasets, we can delineate the unique vulnerabilities of the commercial layer phenotype. While some epidemiological and experimental data suggest that certain broiler lineages may exhibit relative resistance to specific HPAIV strains compared to highly susceptible layer breeds—for instance, demonstrating a higher lethal dose requirement and delayed mortality during the 2015 Midwestern H5N2 outbreak [22,23,24]—susceptibility is a highly dynamic trait that varies widely within each production type depending on the specific genetic lineage, the age of the host at exposure, and the evolutionary adaptation of the challenging virus strain. Furthermore, the apparent absence of infections in certain commercial broiler operations is likely multifactorial. It is driven not only by potential host-intrinsic genetic traits but also heavily by extrinsic variables, such as specific production management systems, shorter lifespan leading to reduced environmental exposure, and stringent biosecurity practices [24]. Conversely, data indicates that the isolation rate of H7N9 in layers has increased annually in China [25,26,27]. This geographic concentration reflects the epidemiological history of H7N9 rather than a region-specific host response, and experimental studies conducted outside China have reported similar susceptibility patterns in layer chickens [28]. Most controlled studies employ specific commercial lines (commonly White Leghorn layers and fast-growing broilers), and differences among international production systems may influence exposure dynamics. The specific mechanisms underlying these breed-specific differences—potentially involving gene polymorphisms and distinct selective pressures [17,25,29,30,31,32,33,34]—remain poorly understood.

**Figure 1:**
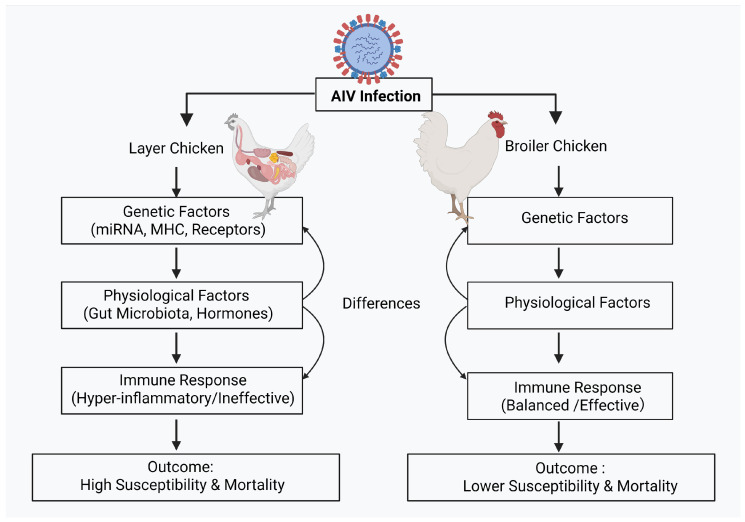
Overview of AIV susceptibility in layer vs. broiler chicken. Created in BioRender. Huang, L. (2026). https://BioRender.com/0dzxztw (accessed on 8 March 2026). Note: The pathways depicted represent generalized experimental observations. Actual disease outcomes may vary significantly depending on bird age, specific breed lineage, and the viral strain.

This review synthesizes the existing literature to elucidate the factors contributing to the differential susceptibility of layer and broiler chickens to AIV. We examine the roles of host factors, including miRNAs, sialic acid (SA) receptors, and gut microbiota, in mediating infection outcomes. By identifying these critical differences, this review aims to provide valuable perspectives on therapeutic interventions and breeding strategies to enhance the protection of susceptible poultry populations. To provide a holistic view of the mechanisms discussed in this review, a schematic framework illustrating the complex interplay of host genetic, physiological, and immunological factors driving the differential susceptibility between layer and broiler chickens is presented in Figure 1.

## 2. Host Determinants Underlying Differential Susceptibility to AIV

The differential susceptibility to AIV observed between poultry breeds is governed by a complex interplay of host factors. Elucidating these determinants is crucial for distinguishing between the mechanisms driving distinct pathogenic outcomes. This section explores key host determinants, ranging from gene regulatory networks (miRNAs) and immunogenetic diversity (MHC) to commensal microbiota and viral receptor specificity.

### 2.1. Differential Expression of MicroRNAs (miRNAs)

MicroRNAs (miRNAs) function as essential post-transcriptional regulators that link host genetic background to antiviral susceptibility. These non-coding RNAs (typically 21–25 nucleotides) [35] orchestrate diverse cellular mechanisms by guiding the cleavage or translational inhibition of target mRNAs [36]. Crucially, they serve as pivotal effectors in host–pathogen interactions, acting as essential regulators of the antiviral response to multiple viruses [37,38,39,40]. Emerging evidence underscores that host genetic background plays a critical role in shaping the miRNA landscape during AIV infection, where immune-related gene expression exhibits significant evolutionary dynamics driven by environmental pressures [41]. Consequently, divergent miRNA profiles constitute a fundamental molecular basis for the variation in susceptibility observed among different poultry breeds and species.

The divergence in AIV susceptibility observed across independent studies of layers and broilers is hypothesized to be underpinned by tissue-specific miRNA signatures that differentially regulate immune and viral gene networks. Research has demonstrated that *gga-miR-34b* and *gga-miR-34c* were significantly upregulated in the trachea of layers infected with the virus compared to non-infected SPF chickens [42]. In contrast, independent transcriptome analysis of infected broiler chickens highlighted the distinct expression profile of *gga-miR-34a*, which was exclusively expressed in the lungs. Notably, bioinformatic analyses and in vitro assays suggest that *gga-miR-34a* targets not only AIV viral genes (*HA*, *NA*, *PA*, *PB1*, and *PB2*) but also a broad spectrum of immune-related host genes, including complement components (e.g., *C1qBP*), apoptosis regulators (e.g., *Caspase-3*), and various interleukins (e.g., *IL-16*, *IL-17RD*). Additionally, specific miRNAs (e.g., *gga-miR-122*, *gga-miR-155*) and immune genes (e.g., *Mx1*, *IL-8*, *IRF-7*) have been identified through expression profiling as strong candidates regulating the host response in broiler lungs. These findings suggest that layers and broilers employ distinct miRNA-mediated strategies to combat viral replication in different anatomical sites [43].

Furthermore, comparative transcriptomics suggests that differential miRNA modulation of the B cell receptor (BCR) signaling pathway may be a contributing factor distinguishing resistant from susceptible hosts. For instance, infection with the H5N1 HPAIV strain (clade 2.3.4) induces rapid mortality (3 dpi) in susceptible SPF chickens (layers), whereas Mallard ducks exhibit delayed mortality (7 dpi) despite receiving the same viral dose [44]. Deep sequencing linked this disparity to opposing miRNA regulation: in the spleen of infected layers, upregulated miRNAs (e.g., *gga-miR-2188-5p*) suppress key genes in the BCR signaling pathway (e.g., *SYK*, *CD81*), potentially impairing antibody production. Conversely, in infected ducks, the downregulation of *apla-miR-122-5p* targets *RASGRP3*, which may enhance BCR signaling, contributing to their resistance [44]. This pattern holds true within chicken genotypes as well; resistant Vietnamese Ri chickens (*Mx/A*; BF2/B21) exhibited elevated levels of *gga-miR-214* and *gga-let-7b* compared to susceptible lines [45]. Thus, the ability to maintain functional immune signaling via specific miRNA regulation is hypothesized to be a hallmark of host resistance. A summary of these differentially expressed miRNAs and their predicted regulatory roles across different breeds is provided in Supplementary Materials Table S1.

Importantly, because direct “head-to-head” miRNA profiling contrasting broilers and layers within the same experimental framework is currently lacking, this table synthesizes findings from independent studies to hypothesize potential breed-specific regulatory networks. Furthermore, it is critical to distinguish between expression profiling and functional causality; many of the miRNA-target interactions discussed above rely on RNA-seq data and computational predictions. In conclusion, while AIV infection disrupts host miRNA homeostasis, which in turn potentially modulates viral tropism and pathogenesis, rigorous in vivo functional validation is required to confirm these regulatory axes. While specific miRNA signatures have been identified, the precise molecular networks by which these non-coding RNAs differentially modulate immune signaling in layers versus broilers remain to be fully characterized. Future research focusing on these breed-specific miRNA-mRNA interactions is essential for developing targeted genetic strategies to enhance disease resistance in susceptible poultry flocks.

### 2.2. Polymorphisms in the Major Histocompatibility Complex (MHC)

Genetic diversity within the Major Histocompatibility Complex (MHC) constitutes a fundamental molecular basis for the differential antiviral immunity observed across poultry breeds. The MHC B-locus, particularly the microsatellite marker *LEI0258*, is strongly associated with susceptibility to avian influenza [46,47,48,49]. While this polymorphism influences immune responses, studies suggest that susceptibility is not solely defined by MHC haplotypes but is also modulated by the broader genetic background of the breed. For instance, experimental infections with H5N1 HPAIV (clade 2.1) in MHC-congenic White Leghorn lines (the predominant commercial layer breed) revealed that strains sharing the same B2 haplotype but differing in background genes exhibited significantly different mortality rates. This indicates that the distinct susceptibility profiles of layers versus other breeds are shaped by a complex interplay between MHC haplotypes and non-MHC background genes [32].

To integrate these line-level observations into a breed-level model, we must consider how artificial selection intersects with background genetics. Intense selection for high egg yield in commercial layers has caused a genetic bottleneck, reducing immunogenetic variability and often fixing specific MHC haplotypes (e.g., the B2 haplotype). In contrast, commercial broiler breeding programs generally maintain broader MHC diversity to support overall robustness. Consequently, the highly specialized background genes selected for maximum reproduction in layers may interact suboptimally with their restricted MHC repertoire during infection. This intersection of production-driven selection and narrowed immunogenetic diversity creates a rigid immune phenotype, fundamentally contributing to the heightened AIV vulnerability of commercial layers compared to broilers.

The capacity to rapidly upregulate MHC class I-mediated cellular immunity distinguishes resistant breeds from certain highly susceptible commercial layer lines. However, it is important to note that immune responsiveness is not uniform across all layer breeds, as it heavily depends on specific MHC haplotypes and the interacting viral strain. The presentation of viral peptides to CD8+ T cells is critical for eliminating infected cells [50]. Comparative studies have shown that Fayoumi chickens (a resistant model) exhibit enhanced MHC class I expression and increased recruitment of T cells in the trachea following H6N2 LPAIV infection. In stark contrast, susceptible White Leghorns failed to mount a comparable local immune response, correlating with higher viral titers in the trachea [51]. This suggests that the high susceptibility often observed in commercial layers is linked to a delayed or suppressed MHC class I response compared to more robust native breeds.

Furthermore, the robust activation of MHC class II-dependent humoral pathways is a key determinant of resistance in specific chicken genotypes. In a study comparing resistant and susceptible lines of Ri chickens infected with H5N1 HPAIV (clade 2.3.2.1), resistance was characterized by the significant upregulation of MHC class II genes (*DMR2*, *BLB2*) and co-stimulatory molecules (*CD80*, *CD86*, *CD40*). The upregulation of *CD40*, which facilitates B cell activation and antibody production, was notably absent or lower in susceptible birds. These findings imply that breed-specific resistance relies on the efficient coordination of antigen presentation and helper T cell signaling, a mechanism that may be less effective in highly susceptible phenotypes [52]. While miRNAs primarily regulate innate immune signaling and antiviral gene expression, MHC polymorphisms predominantly influence antigen presentation and adaptive immune activation, indicating that these determinants may operate at distinct yet complementary levels of host immunity.

### 2.3. Composition and Regulation of Gut Microbiota

The avian gastrointestinal microbiome functions as a critical extrinsic organ that modulates immune development and host health. Comprising a diverse array of bacteria, viruses [53], and fungi [54], the gut microbiota is indispensable for physiological maturation, particularly the establishment of the avian immune system [55,56,57]. A balanced microbial community confers protection through multiple mechanisms: probiotics enhance mucosal immunity by promoting anti-inflammatory cytokine secretion and activating dendritic cells [58,59]. Conversely, the invasion of pathogens can induce dysbiosis—the destruction of beneficial commensals—which disrupts the mucosal barrier and upregulates virulence genes, thereby accelerating host debilitation [60,61].

Host genetic background exerts a profound influence on the composition of the gut microbiota, leading to distinct microbial profiles between layers and broilers. While environmental factors are impactful, breed-specific genetic traits significantly shape the diversity and abundance of intestinal commensals. A comparative study employing 16S rRNA sequencing and metagenomics revealed marked differences in the cecal microbiota structure of these two breeds. Notably, the abundance of *Desulfovibrio* was significantly higher in the cecum of layers compared to broilers. Given that specific bacterial taxa can modulate immune responses, these breed-specific microbial differences may constitute a potential environmental factor driving the differential susceptibility to infection observed between layers and broilers [62].

Furthermore, the disruption of gut homeostasis is closely linked to the pathogenesis of intestinal and systemic diseases. Pathogenic bacteria and the toxins they produce can directly damage intestinal epithelial cells, impair nutrient absorption, and trigger immunosuppression or excessive inflammation, all of which impede chicken growth [63]. Crucially, the interplay between the gut microbiome and the host immune system plays a decisive role in determining the outcome of viral infections. By affecting gut morphology and immune responses, the distinct microbiome profiles of layers and broilers likely contribute to their varying resilience against pathogens. Although direct experimental models comparing AIV infection outcomes based solely on layer versus broiler microbiomes are currently lacking, these baseline microbial differences provide a strong rationale for future comparative investigations [53,61,64]. This suggests that modulating the microbiota could be a viable strategy to enhance resistance.

### 2.4. Distribution Patterns of SA Receptors

The specific binding affinity between viral hemagglutinin and host SA receptors constitutes the primary determinant of tissue tropism and host susceptibility. Influenza viruses initiate infection by binding to cell surface glycans: avian viruses preferentially bind α-2,3-linked N-acetylneuraminic acid (α-2,3SA), while human strains prefer α-2,6-linked forms (α-2,6SA) [65,66]. The density and distribution of these receptors function as a biological “gatekeeper,” dictating the severity of infection. Although AIV demonstrates broad tissue tropism [21,67], clinical outcomes vary drastically among species due to receptor heterogeneity. For instance, while infection in ducks typically results in mild or asymptomatic shedding, similar infections in chickens can accelerate viral evolution and cause high mortality [68]. Thus, mapping these receptor landscapes is crucial for understanding why commercial layers exhibit distinct vulnerability compared to other avian hosts.

Comparative analysis of the gastrointestinal tract reveals that commercial layers possess a significantly broader and denser receptor distribution than waterfowl. Immunohistochemical studies indicate that while the tracheal epithelium of White Leghorn chickens, Pekin ducks, and turkeys all show strong positivity for avian-type α-2,3SA receptors, striking differences emerge in the gut. In ducks and turkeys, α-2,6SA expression is minimal in the intestines. In stark contrast, White Leghorn chickens exhibit high levels of both α-2,3SA (50–80%) and α-2,6SA (20–50%) receptors throughout the small and large intestinal epithelium. This extensive “dual-receptor” profile in the gut suggests that layers are anatomically predisposed to more efficient enteric viral replication and transmission compared to waterfowl [69].

While these inter-species comparisons establish the baseline susceptibility of chickens, it is important to clarify that direct, side-by-side experimental quantification of SA receptor densities specifically between commercial layers and commercial broilers is currently lacking in the literature. Based on differences in systemic viral dissemination and generalized observations, broilers are inferred to possess a less extensive enteric and systemic receptor profile compared to highly susceptible layer lines; however, this remains a critical gap requiring future quantitative research.

**Figure 2:**
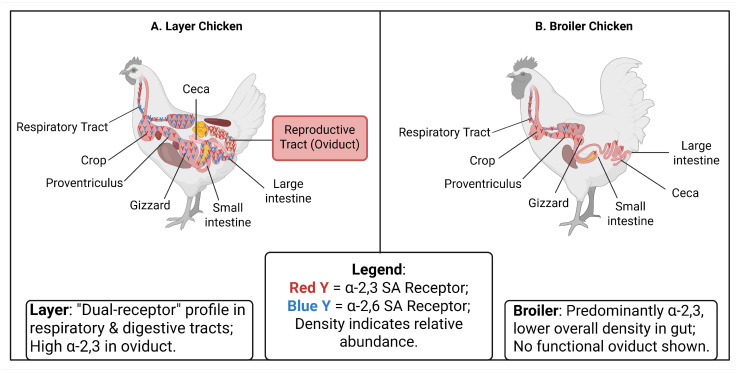
Comparative SA receptor distribution in layer vs. broiler Chicken. Created in BioRender. Huang, L. (2026). https://BioRender.com/hjh2thl (accessed on 8 March 2026).

Based on this current understanding, the distinct anatomical landscapes of α-2,3SA and α-2,6SA receptors, highlighting the extensive “dual-receptor” profile in the layer respiratory and gastrointestinal tracts compared to the broilers, are visualized in Figure 2.

Transitioning to intra-species comparisons, intrabreed studies further demonstrate that commercial layers possess a “hyper-susceptible” receptor profile in the respiratory tract and immune cells compared to indigenous breeds. Studies contrasting White Leghorns (susceptible layers) with Silky Fowl (a resistant indigenous breed) show that Silky Fowl express limited α-2,3SA and no detectable α-2,6SA in the tracheal mucosa. Conversely, the presence of both receptor types in Leghorn tracheas increases their potential to act as “mixing vessels” for diverse viruses. Beyond the epithelium, receptor expression on immune cells dictates systemic spread. The cecal lamina propria of Silky Fowl shows markedly lower receptor levels and reduced numbers of macrophages (F4/80+) and T cells (CD3+) compared to Leghorns. Given that AIV can directly infect macrophages [67,70], the high receptor density on immune cells in Leghorns may facilitate a “Trojan horse” mechanism, aiding systemic viral dissemination, whereas the reduced expression in Silky Fowl contributes to their relative resistance [71,72].

A unique feature contributing to the severe economic impact of layers is the abundant expression of AIV receptors in the reproductive tract. HPAIV infections (e.g., H5N2, H7N7) frequently cause systemic disease and drastic drops in egg production [73,74,75]. This is mechanistically linked to the specific enrichment of α-2,3SA receptors in the oviduct of layers, whereas α-2,6SA receptors are rarely detected (Figure 2) [76,77]. Crucially, the density of these receptors in the reproductive tract correlates positively with viral load and pathological severity. This receptor distribution renders the layer reproductive system a primary target for viral replication, distinguishing the pathogenesis in layers from that in non-laying breeds [78].

## 3. Hormones Regulate Host Genes to Affect Viral Dynamics

Hormonal fluctuations function as systemic modulators of the immune landscape, directly influencing host susceptibility and viral pathogenesis. The onset of viral infection triggers alterations in neuroendocrine levels, which in turn regulate a wide range of physiological processes, including the intrinsic antiviral response [79,80,81,82,83,84,85,86]. Sex steroid hormones, in particular, have been recognized as potent regulators of adaptive immunity, capable of upregulating or downregulating humoral responses depending on the host’s physiological state [87,88,89,90,91]. Consequently, the endocrine status of the host serves as a critical variable in determining the outcome of influenza virus infections.

Experimental evidence from mammalian models suggests that sex steroids differentially modulate inflammatory severity, a mechanism likely conserved in avian species. Studies have demonstrated that androgens (e.g., testosterone) generally attenuate proinflammatory cytokine responses, potentially protecting against severe lung pathology [92]. For instance, lower pre-infection plasma testosterone levels were associated with increased viral load and cytokine storms in H7N9-infected hosts [93]. Conversely, estrogens and progesterone exhibit complex immunomodulatory effects; while they recruit immune cells, dysregulated levels can exacerbate inflammation [93,94,95,96,97,98,99,100,101,102] (Table 1).

Previous research indicates that exposure to H9N2 LPAIV elevates plasma glucocorticoids, which subsequently suppress pulmonary antiviral responses [103,104]. Thus, the distinct hormonal milieus of males versus females, or layers versus broilers, may underpin the observed variances in disease severity.

Crucially, the physiological divergence between layers and broilers creates distinct hormonal environments that may drive breed-specific susceptibility. Layers are metabolically prioritized for reproduction, characterized by significant surges in estrogen and progesterone, particularly during peak egg-laying periods [105]. In contrast, broilers are selected for rapid somatic growth, exhibiting distinct profiles of metabolic hormones such as triiodothyronine (T3) and thyroxine (T4) [106]. This divergence reflects long-term artificial selection pressures: reproductive endocrine dominance in layers versus growth–metabolic axis prioritization in broilers. Consequently, endocrine variation between breeds is intrinsically linked to production traits, with reproductive hormones predominating in egg-laying hens and anabolic/metabolic hormones predominating in meat-type birds. It is hypothesized that the high-estrogen environment in layers, essential for egg production, may inadvertently enhance susceptibility to AIV or exacerbate immunopathology compared to the growth-centric hormonal profile of broilers. A reduction in hormone concentrations is often linked to the shifting metabolic demands between reproductive activities and growth [18]. While adaptive immune responses (e.g., macrophage and lymphocyte recruitment) differ between resistant and susceptible breeds [51,107], the upstream hormonal drivers of these immune variances remain a critical knowledge gap. However, direct comparative endocrine profiling between layer and broiler chickens in the context of AIV infection remains limited, and correlations between specific hormone levels and viral replication dynamics have not been systematically established. Addressing this gap represents an important direction for future research.

In summary, the interplay between the reproductive endocrine cycle and antiviral immunity represents a significant but understudied factor in poultry pathology. Current research has yet to fully elucidate whether the peak-laying hormonal surge directly predisposes layers to higher mortality rates compared to non-laying broilers. Future investigations linking specific hormone levels to viral replication kinetics are essential for unraveling the mechanisms of breed-dependent susceptibility.

## 4. Age May Account for the Differential Manifestation of AIV

The ontogeny of the avian immune system significantly dictates the robustness of host responses, making age a critical variable in AIV susceptibility. Developmental disparities in the transcriptional landscape of immune genes are evident between juvenile and mature birds. For instance, following H9N2 LPAIV infection, 1-week-old birds exhibited a markedly blunted immune response, characterized by fewer stimulated immune-related genes compared to older cohorts. This reduced responsiveness is attributed to the functional immaturity of antigen-presenting cells, T cells, and NK cells in naïve birds [108]. Consequently, the developmental stage of the host directly limits the magnitude of transcriptional defense mechanisms, rendering younger birds potentially more vulnerable to viral replication.

However, in the context of commercial poultry, “age” is intrinsically linked to the distinct production lifespans of layers and broilers, complicating direct breed comparisons. Studies indicate that adult birds mount a significantly more potent secondary antibody response compared to juveniles [109]. This is particularly relevant for layers, which are reared for extended production cycles (often >70 weeks) compared to the short lifespan of broilers (<8 weeks). While broilers are susceptible to HPAIV infection regardless of age, their short lifespan means they are typically infected while their immune systems are still developing. In contrast, layers face AIV risks across varied physiological stages, including peak lay and potential immunosenescence. Currently, a significant knowledge gap exists regarding whether the “age factor” independently modulates susceptibility in adult layers versus broilers, or if it acts purely as a function of exposure time. Therefore, the apparent susceptibility of layers may be significantly confounded by their longer lifespan. From an epidemiological perspective, an extended production cycle (often >70 weeks) inherently increases the cumulative statistical probability of environmental exposure to diverse AIV strains and seasonal spillover events. More importantly, this prolonged lifespan inevitably subjects layers to age-related immune decline (immunosenescence). As these birds age, progressive deterioration of immune function—such as reduced lymphocyte diversity and impaired antigen-presenting capabilities—diminishes their ability to mount or sustain effective antiviral responses. Consequently, what is often interpreted as an intrinsic, breed-specific “hyper-susceptibility” in field observations may partially reflect these temporal dynamics: a vastly longer exposure window coupled with a naturally waning immune system, a physiological endpoint that short-lived broilers (<8 weeks) do not reach.

## 5. Discussion

The reported disparities in AIV infection outcomes associated with different poultry production types represent a critical intersection of host genetics, physiology, and viral ecology. While viral evolution is often the primary focus of influenza research, this review highlights that host-intrinsic factors are equally important in determining pathogenesis. While experimental evidence frequently highlights heightened vulnerability in specific layer lines—characterized by systemic viral replication and severe egg production declines—this susceptibility is unlikely to be attributable to a single determinant. It instead reflects the combined effects of genetic regulation, receptor distribution, and reproductive physiology. Crucially, it must be emphasized that susceptibility is not an absolute or universal trait of all layers, nor is resistance guaranteed for all broilers. Infection outcomes vary widely within each production type depending on the host’s age at exposure, the specific breed lineage, and the pathogenicity of the challenging virus strain.

At the molecular level, differences in gene regulation may contribute to breed-specific immune responses. Although host genetics influence resistance [110], recent findings emphasize the role of non-coding RNA networks. Differential miRNA expression between layers and broilers suggests that regulatory variation may influence antiviral pathways, including B cell receptor signaling and interferon activation. Furthermore, although MHC haplotypes are central to antigen presentation, evidence that resistance often depends on non-MHC background genes indicates that post-transcriptional regulatory mechanisms may modulate the overall efficiency of adaptive immunity. Future studies should prioritize functional validation of specific miRNA–mRNA interactions and clarify whether these pathways modify MHC-mediated antigen presentation.

At the anatomical level, sialic acid (SA) receptor distribution influences viral entry and tissue tropism. Compared with broilers or waterfowl, layers exhibit broader expression of both α-2,3SA and α-2,6SA receptors across respiratory and gastrointestinal tissues. Receptor enrichment in the layer oviduct correlates with reductions in egg production during infection [78]. Such distribution patterns may increase the likelihood of systemic dissemination and reassortment, although direct comparative quantification between production types remains limited.

At the physiological level, divergence in susceptibility may reflect trade-offs associated with production selection. The “cost of reproduction” hypothesis suggests that the hormonal surge (estrogen/progesterone) required for peak egg laying in layers may inadvertently induce a transient immunosuppressive state or alter tissue permeability [105]. This stands in sharp contrast to broilers, which are metabolically prioritized for somatic growth and typically do not reach reproductive maturity before harvest. Additionally, the gut microbiota, acting as an extrinsic immune organ, differs significantly between these breeds. Given that layers are more prone to enteric infection, dysbiosis in the layer gut—potentially driven by distinct genetic or hormonal profiles—may compromise mucosal barriers (Toll-like receptors/Type I Interferons) that remain robust in broilers [11,111]. However, direct comparative evidence linking microbiota composition to Toll-like receptors/Type I interferon signaling and influenza outcomes between layers and broilers remains limited. Further mechanistic studies are required to determine whether microbiota-driven modulation of innate immune pathways contributes to breed-specific differences in AIV susceptibility.

## 6. Conclusions

In summary, the varying susceptibility to AIV observed between layer and broiler chickens is not attributable to a single biological factor but is a multifaceted phenomenon driven by the interplay of receptor density, hormonal cycles, and gene regulation. Under certain infectious conditions, specific commercial layer lines can exhibit a convergence of multiple physiological and molecular vulnerabilities: a high density of SA receptors in the reproductive tract, a hormonal environment prioritized for egg production that may dampen immunity, and specific miRNA profiles that suppress antiviral signaling. To advance poultry protection, future strategies must move beyond generic vaccination. Research should prioritize: (1) elucidating the specific mechanisms by which reproductive hormones modulate host antiviral immunity to understand the cost of reproduction; (2) microbiome Engineering to explore if “broiler-type” microbiota can confer resistance; and (3) molecular Breeding utilizing specific miRNA signatures to select for high-yielding layers with enhanced innate resistance. Unraveling these complex host–pathogen interactions is essential for developing next-generation breeding strategies tailored to the unique physiological demands of the layer industry.

## Figures and Tables

**Table 1:** Effects of hormones on immune response to influenza A virus infection.

Species	Virus Subtypes	Hormones	Functions (Effects on Immune Response)	References
Mice	H7N9	Testosterone	Infection depletes plasma levels; low testosterone correlates with high viral load and testicular inflammation.	[93]
Mice	H1N1	Testosterone	Enhances survival; depletion correlates with increased severity in aged hosts.	[94]
Mice	H1N1	Testosterone	Attenuates lung inflammation and resolves leukocytes; enhances CD8+ T cells; viral load unaffected.	[95]
Mice	H1N1	Estradiol	High doses reduce lung TNF-α/CCL2 (≥10-fold) and improve female survival via ERα.	[96]
Mice	H5N1	Estradiol	Delays specific antibody production; reduces IgG- and IL-4-secreting cells, promoting an anti-inflammatory state.	[97]
Mice	H1N1	Estriol	Likely mimics estradiol effects via Estrogen Receptor (ER) signaling.	[98]
Mice	H1N1	Progesterone	Promotes lung repair (via AREG upregulation) and recovery; increases TGF-β and Treg cells without affecting viral load.	[99]
Mice	H1N1	Progesterone	Protects against primary infection but exacerbates immunopathology during secondary heterologous infection.	[100]
Shorebird	H5N1	Corticosterone	Modulates immunity during migration; potentially increases susceptibility (mechanism unclear).	[101]
Human	H7N9	Cortisol	Infection correlates with elevated cortisol and reduced cellular immunity.	[102]

## Data Availability

No new data were created or analyzed in this study. Data sharing is not applicable to this article.

## Supplementary Materials

The following supporting information can be downloaded at: https://www.mdpi.com/article/10.3390/cimb48040359/s1.
